# Pasteurellosis in camels in Southern Mongolia: A case report

**DOI:** 10.29374/2527-2179.bjvm000624

**Published:** 2024-10-07

**Authors:** Myagmar Erdenechimeg, Gurdorj Soyolmaa, Guofeng Cheng, Tsogtsaikhan Bayarsaikhan, Altangerel Tsogtsaikhan Dursahinhan, Tseveenjav Lundaa, Tserendorj Munkhjargal

**Affiliations:** 1 Veterinarian, School of Veterinary Medicine, Mongolian University of Life Sciences, Zaisan–17024, Ulaanbaatar, Mongolia; 2 Veterinarian, Department of Veterinary Services, Umnugobi province, Mongolia; 3 Veterinarian, Institute of Veterinary Medicine, Mongolian University of Life Science, Zaisan–17042, Ulaanbaatar, Mongolia; 4 Veterinarian, Shanghai Tenth People's Hospital, Institute for Infectious Diseases and Vaccine Development, Tongji University School of Medicine, Shanghai, China; 5 Biologist, H.W. Manter Laboratory of Parasitology, University of Nebraska State Museum, University of Nebraska-Lincoln, USA

**Keywords:** Pasteurella multocida, Hemorrhagic septicemia, PCR, Umnugobi, Pasteurella multocida, Septicemia hemorrágica, PCR, Umnugobi

## Abstract

Hemorrhagic septicemia (pasteurellosis) in animals, caused by *Pasteurella multocida* Trevisan 1887, is a significant but previously undocumented disease in Mongolian camels. *Pasteurella multocida*, a small Gram-negative coccobacillus, typically exists commensal in the nasopharynx of camels but can cause severe illness under certain environmental stressors. This study reports the first case of cameline hemorrhagic septicemia in Gobi region of Mongolia, specifically in Umnugobi province, where acute septicemia affected 26 camels, resulting in 10 deaths within 24-48 hours. Clinical signs included depression, inappetence, lethargy, increased rectal temperature, and paralysis of the lower lip. Surviving camels responded to treatment with Lactate Ringer solution and antibiotics. Postmortem examinations revealed acute pulmonary congestion and necrotic liver. Molecular diagnostic test, PCR, confirmed the presence of *P. multocida* with the identification of the *KMT1* gene. This case underscores the potential for significant economic losses due to hemorrhagic septicemia in camels and highlights the need for early detection and treatment to mitigate its impact. The initial attempt at implementing a vaccination program effectively controlled the potential further outbreak. This study emphasizes the importance of continuous surveillance and preventive measures in managing hemorrhagic septicemia in livestock.

## Resumo

A septicemia hemorrágica (pasteurelose) em animais, causada *por Pasteurella multocida* Trevisan 1887, é uma doença significativa, mas anteriormente não documentada, em camelos mongóis. *Pasteurella multocida*, um pequeno cocobacilo Gram-negativo, normalmente existe como comensal na nasofaringe de camelos, mas pode causar doenças graves sob certos estressores ambientais. Este estudo relata o primeiro caso de septicemia hemorrágica de camelos na região de Gobi, na Mongólia, especificamente na província de Umnugobi, onde a septicemia aguda afetou 26 camelos, resultando em 10 mortes em 24-48 horas. Os sinais clínicos incluíram depressão, inapetência, letargia, aumento da temperatura retal e paralisia do lábio inferior. Os camelos sobreviventes responderam ao tratamento com solução de Lactato Ringer e antibióticos. Os exames post-mortem revelaram congestão pulmonar aguda e fígado necrótico. O teste de diagnóstico molecular, PCR, confirmou a presença de *P. multocida* com a identificação do gene KMT1. Este caso sublinha o potencial de perdas económicas significativas devido à septicemia hemorrágica em camelos e destaca a necessidade de detecção e tratamento precoces para mitigar o seu impacto. A tentativa inicial de implementar um programa de vacinação controlou eficazmente o potencial novo surto. Este estudo enfatiza a importância da vigilância contínua e de medidas preventivas no manejo da septicemia hemorrágica na pecuária.

## Introduction

Hemorrhagic septicemia (pasteurellosis) is caused by *Pasteurella multocida* (*P. multocida*), a small Gram–negative coccobacillus that is commensal in the nasopharynx of camels. *P. multocida* strains express a polysaccharide capsule on their cell surfaces. They are classified into three subspecies (*multocida*, *septica*, and *gallicida*), A, B, D, E, and F capsular types and are further subtyped into 16 serovars (Harper et al., 2006).

Although *P. multocida* is a part of the normal respiratory microbiota in camels, it becomes pathogenic and causes the disease when harmful environmental influences lower the resistance of the camel body. The morbidity of pasteurellosis is low, but mortality is high. Carrier or sick camels are considered as source of infection to other animals. Pasteurellosis leads to the manifestation of severe clinical signs, such as fever, pulmonary edema, fibrinous pneumonia, and diarrhea, and death usually occurs within 2-3 days (De Alwis, 1992).

In 1950, the first case of hemorrhagic septicemia in cattle in Mongolia was recorded. In 1978, researchers started to study on prevalence, treatment, and prevention of hemorrhagic septicemia. Mainly, hemorrhagic septicemia caused by *P. multocida* type A and B in cattle, yak, and sheep is still among the most economically important disease of domestic animals in Mongolia (Lundaa et al., 2009). However, this disease has not been reported yet in camels. In the present study, we reported the first case of cameline hemorrhagic septicemia was caused by *P. multocida* in Mongolia.

## Case report

Umnugobi province (43°0’N, 104°15’E) is in Southern Mongolia (Supplementary Figure 1), and there is the largest camel population (164.8 thousand), accounting for over 30% of the country (Mongolian Statistical Information Service, 2024). In September 2019 and August 2020, cases of acute septicemia occurred in free-range domestic animals, including Mongolian camels, *Camelus bactrianus* Linnaeus, 1758, in Khanbogd and Bayan-Ovoo soums, Umnugovi province. The acute septicemia affected 26 adult camels. The most characteristic findings in sick camels were marked depression, inappetence, lethargy, recumbency, increased rectal temperature (39-40°C), salivation, and paralysis of the lower lip. Ten out of the affected camels died within 24-48 hours. The herd contained 5,995 animals, including 70 cattle, 380 sheep, 420 goats, and 5,125 camels, none of which got sick. Their feeding was pasture-based, and drinking water was provided by a deep well from a natural source. This water source was also frequented by local wildlife, such as wild Bactrian camels, *Camelus ferus* Przewalski, 1878, and other domestic animals.

The remaining 16 sick camels recovered after the initiation of intravenous Lactate Ringer solution at 80 ml/kg/day and a single dose of 1 ml/20 kg body weight of Calcium, Magnesium, and Penoksal-La, along with 30 ml of multivitamin injected intramuscularly for three days following the first detection of clinical signs. Camels in the area were vaccinated with a *P. multocida* vaccine (Biocombinat state-owned factory, Mongolia) which is used in Mongolia for protecting cattle and sheep. Khanbogd and Bayan-Ovoo soums had been free of hemorrhagic septicemia until this point. A total of 5125 camels were vaccinated for the first time. Deceased animals were subjected to post-mortem examinations at the Department of Veterinary Services in Umnugobi province and the Institute of Veterinary Medicine in Mongolia for necropsy, as well as histopathological, microbiological, and molecular analyses.

**Figure 1:**
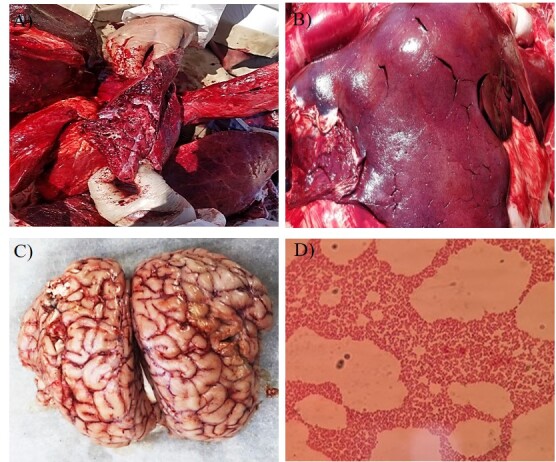
A) Haemorrhages in the lung, B) liver, and C) congestion of the central nervous system. D) Gram negative, cocco-bacillary or rod shaped *Pasteurella multocida* in Gram staining (100x objects).

**Figure 2:**
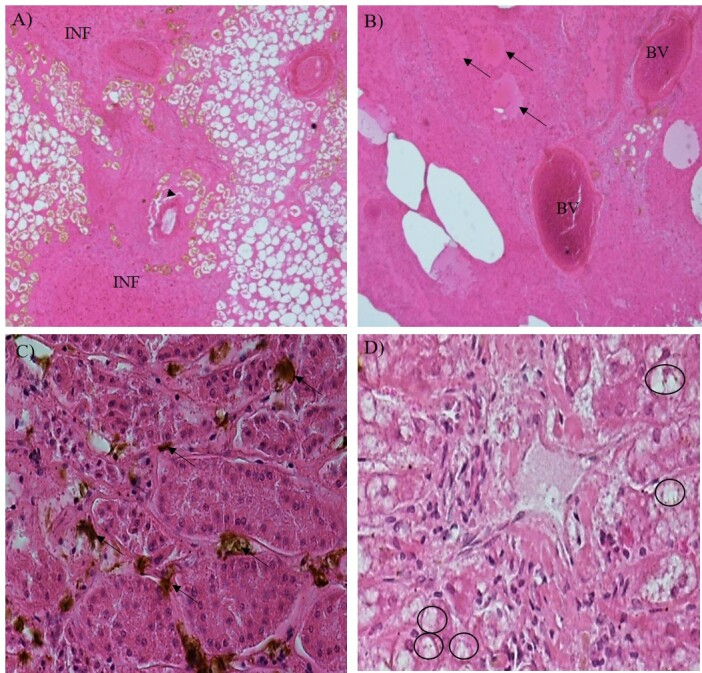
Histopathological lesions in camel’s organs: A and B) Alveolar edema (arrow) and bacterial foci (INF) in the lung НЕ, х40, and x400, respectively. C) Hemosiderin (arrow) accumulation in the kidney and D) fatty degeneration (black circles) of the liver НЕ, х400.

During the standard procedural examination, acute congestion in the lungs and central nervous system, and hemorrhages in the livers were observed in all the animals (Figure 1A-C). The trachea was shown to be congested containing frothy fluid. Also, blood-tinged fluid was found in the thoracic and abdominal cavities. Tissue samples from the lung, liver, spleen, kidney, heart, thymus, and retropharyngeal lymph nodes of five animals were collected after necropsy for histopathological and microbiological studies. The samples were fixed in 10% neutral-buffered formalin, embedded in paraffin, sectioned into 5 μm sections, and stained with hematoxylin and eosin (HE) for the histopathological study. The lung tissue samples showed the evidence of alveolar edema, bacterial foci, and severe congestion (Figure 2A and B). The image revealed calcium salt deposition with hemosiderin pigment in the kidney tissue and fatty degeneration in the liver tissue (Figure 2C and D, respectively).

Microbiological analyses involved aseptically collected samples from specified organs, which were plated on blood agar, Tryptic Soy Agar (Bacto, USA), and MacConkey’s Agar (Sigma-Aldrich, USA). The plates were then incubated at 37°C for 24–48 hours under microaerobic conditions. Isolated bacterial samples were cultured on Tryptic Soy Broth (TSB-Bacto, USA) and incubated at 37°C for 18 hours. The EB-20 test (Nissui Pharmaceutical Co. Ltd., Tokyo, Japan) was inoculated, incubated at 37°C for 18-20 hours, and interpreted as recommended by the manufacturer. Based on the morphological examination of the colony using Gram staining (Gram-negative coccobacilli, see Figure 1D) and biochemical reactions, the presence of *P. multocida* was confirmed with an identification accuracy of 99% (Table 1). This marks the initial successful identification of P. multocida in camels.

**Table 1:** Results of biochemical identification of the isolated *Pasteurella multocida* using standard laboratory testes

**Results**	**Biochemical tests**
-ve	Hemolysis on blood agar
-ve	MacConkey agar growth
-ve	Hydrogen sulifide
-ve	Esculin
-ve	PPA
+ve	Indole
+ve	Sodium Pyruvate
-ve	Trisodium citrate
-ve	Lysine hydrochloride
-ve	Arginine hydrochloride
+ve	Ornithine hydrochloride
-ve	2-Nitrophenyl ß-D-Galactopyranoside
-ve	Urea
-ve	Disodium Malonate
-ve	Adonite
-ve	Inositol
-ve	Raffinose
-ve	Rhamnose
+ve	Sorbitol
+ve	Sucrose
+ve	Mannitol
-ve	Arabinose

The identification was further confirmed by detecting the *KMT1* gene using a species-specific PCR assay. *P. multocida* isolates were identified by PCR, and *KMT1* gene was amplified at 460 bp.

**Figure 3:**
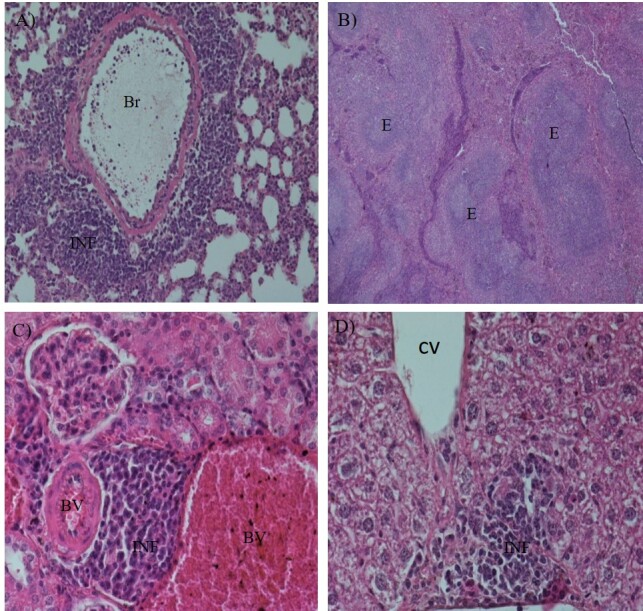
A) Hemorrhagic pathological changes of the lung (HE, x200), B) spleen (HE, x40), C) kidney (HE, x400), and D) liver (HE, x100) of mice experimentally infected with *Pasteurella multocida*. Br- brochial, INF- inflammatory cells, E-edema, BV- blood vessels, CV- central vein.

Furthermore, mice bioassay revealed that *P. multocida* isolate was highly pathogenic to BALB/c mice with a mean death time of 24-48 hours with post-mortem finding of septicemia (Figure 3A-D) and *P. multocida* was reisolated from infected organs and showing Gram-negative coccobacilli by Gram stain. Further confirmation was conducted by PCR.

## Discussion

Pasteurellosis is a disease of significant economic importance in Mongolian livestock. Hemorrhagic septicemia affects not only cattle, yaks, sheep, and goats, but also, in some cases, horses, pigs, and wild animals. Younger animals appear to be more susceptible to this pathogen. In Mongolia, the incidence of the disease is highest in high mountain areas during the winter and spring seasons. Similarly, the frequency of infection was significantly higher in winter, and spring as compared to autumn and summer in Iraq and Ethiopia (Alemneh & Tewodros, 2016; Rasha et al., 2014). It may be due to the high rain fall of the area in winter and spring which exposes the animals to cold stress and immune-compromization.

Hemorrhagic septicemia had affected sheep in 16 of the 21 provinces of Mongolia between 1970 and 1988. Moreover, during 1975s and 2000s epizootics occurred in 239 soums of 21 provinces, Mongolia, and the mortality rate due to hemorrhage septicemia was 8.4% in cattle. With the adaptation of vaccines and drugs, the incidence of disease and the mortality rate have been reduced considerably.

Biocombinat state-owned factory of Mongolia manufactures vaccines for Hemorrhagic septicemia. In the vaccine development process, a virulent *P. multocida* strain is inactivated by formaldehyde and adjuvanted with aluminum hydroxide gel. This vaccine was used to administer to a total of 5125 camels in Khanbogd and Bayan-Ovoo soums in the Umnugovi province for the prevention of hemorrhagic septicemia.

The diagnosis of pasteurellosis relies on clinical signs, gross pathological lesions, isolation of pathogens, and biochemical and molecular characterizations. The microbiological and biochemical features of the isolates, along with the clinical presentation observed in affected camels in this study, are consistent with *P. multocida*. Specifically, acute symptoms such as depression, inappetence, lethargy, recumbency, high temperature (40°C), salivation, and paralysis of the lips closely resemble those of hemorrhagic septicemia. Furthermore, in the pathogenicity test, the *P. multocida* isolate exhibited high pathogenicity. These findings are consistent with previous studies by OIE Terrestrial (2008), and Hassan and Mustafa (1985).

PCR technology enables rapid, sensitive, and specific detection of these pathogens; *P. multocida*-specific PCRs can identify all subspecies of *P. multocida*. In this study, *P. multocida* isolates were identified using PCR with specific primers targeting the *KMT1* gene. Similarly, researchers in Sudan, Iran, Kenya, and Egypt have also utilized KMT1SP6-KMT1T7 primers to detect *P. multocida* infections in camels (Dutta et al., 2001; Hanafy et al., 2022; Hassan & Mustafa, 1985; Kasivalu et al., 2022; Tahamtan et al., 2016).

To our knowledge, this disease has not been previously reported in the cameline species in Mongolia. Therefore, this is the first reported case to describe the characteristics of acute hemorrhagic septicemia in camels in Mongolia. Although the source of infection could not be accurately determined, it may be linked to contact with infected cattle, sheep, or wildlife hosts or shared contaminted resources in the area.

In this study, we observed low morbidity (0.5%) and mortality (38.5%) rates in camels. Similar low rates of morbidity and mortality have been reported in wild boar (Risco et al., 2013), swine (Ujvári et al., 2015), and cattle (Magyar et al., 2017). In contrast, Cardoso-Toset et al. (2013) reported high morbidity (70%) and mortality (95%) rates in swine. These variations may stem from differences in host species, timing of treatment administration, and other factors (Shivachandra et al., 2011). Moreover, early antibiotic treatment could effectively increase recovery rates and minimize losses.

## Conclusions

This study represents the first documented case of acute hemorrhagic septicemia caused by *P. multocida* in camels within Mongolia. Previously associated primarily with cattle, yaks, and sheep in Mongolia, this disease has now been identified in camels, highlighting its potential impact on camel health and the broader livestock industry in the region. The clinical signs observed, including depression, inappetence, lethargy, and high fever, closely resemble those seen in other affected species.

Diagnosis was confirmed through a comprehensive approach including clinical observation, gross pathological findings, microbiological assays, and molecular techniques, all consistently identifying *P. multocida* as the causative agent. The severity of symptoms and mortality rates observed underscore the urgent need for effective prevention and treatment strategies in camel populations.

The vaccination campaign launched in response to this outbreak, utilizing a formaldehyde-inactivated *P. multocida* vaccine adjuvanted with aluminum hydroxide gel, marks a critical step in controlling the spread of hemorrhagic septicemia among camels in Umnugovi province. This proactive approach, coupled with early antibiotic intervention, has demonstrated efficacy in reducing morbidity and mortality rates.

Future research efforts should prioritize understanding the epidemiology of *P. multocida* infections in Mongolian camels, including identifying potential reservoirs and transmission modes. Continued surveillance and periodic vaccination campaigns will be crucial for mitigating the impact of hemorrhagic septicemia on camel health and livelihoods in Mongolia.

## Supplementary Material

Supplementary material accompanies this paper.

Supplementary Figure 1. Geographical maps of Umnugobi province and Mongolia. Cameline tissue samples were collected from the Bayan-Ovoo and Khanbogd soums (shown in red type) of Umnugobi province, Mongolia.

This material is available as part of the online article from http://doi.org/10.29374/2527-2179.bjvm000624
