# Supplementary figures and images for: Pasteurellosis in camels in Southern Mongolia: A case report
Source: Braz J Vet Med. 2024 Oct 7;46:e000624. doi: 10.29374/2527-2179.bjvm000624 (PMC11466242; doi:10.29374/2527-2179.bjvm000624)

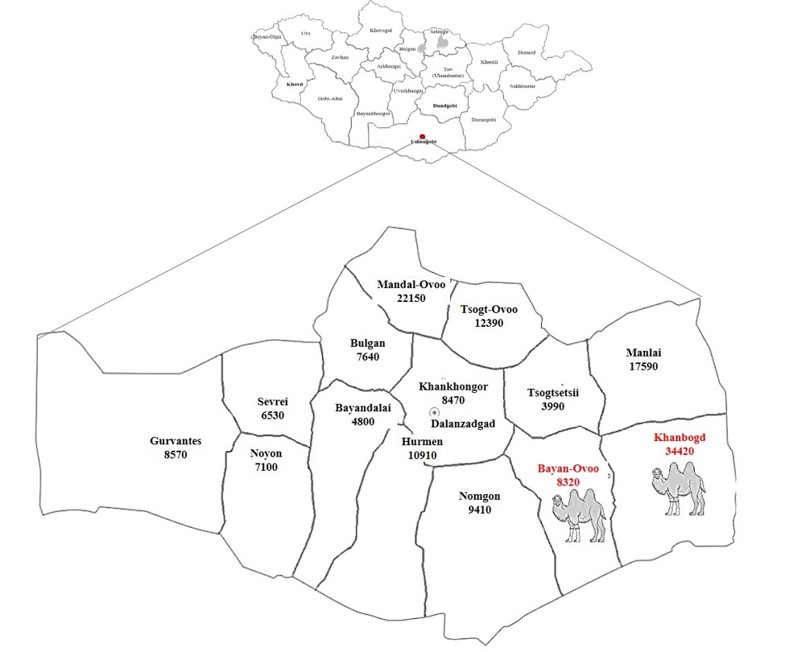

Supplement: Supplementary Figure 1. [file bjvm-46-e000624-Suppl01.jpg]
